# Redefining online biology education: a study on interactive branched video utilisation and student learning experiences

**DOI:** 10.1002/2211-5463.13767

**Published:** 2024-01-18

**Authors:** Melissa M. Lacey, Nigel J. Francis, David P. Smith

**Affiliations:** ^1^ Department of Biosciences and Chemistry Sheffield Hallam University Sheffield UK; ^2^ School of Biosciences Cardiff University Cardiff UK

**Keywords:** active learning, adventure learning, online content, problem‐solving, student‐led learning, videos

## Abstract

This study evaluated the use of interactive, branched videos compared with traditional passive linear delivery for enhancing student engagement and learning in online courses. Undergraduate biology students were provided with either branched decision‐based or linear videos on cell biology and protein purification as self‐guided or consolidation activities. While the interactive branched videos did not improve learning gains, thematic analysis revealed that students found them more enjoyable and preferable for revision. However, most students felt linear passive videos were more logically structured for core content delivery. In a revised format, with clearer scaffolding, the interactive branched videos were perceived as significantly more engaging and useful when utilised for a problem‐solving activity. Students welcomed the autonomy of directing their learning path but desired support to avoid missing critical information. Overall, thoughtfully designed branched videos can increase student motivation, but their utility depends on context. Our findings indicate the importance of balancing interactivity, clear organisation and purpose when incorporating these innovative formats into online learning. Branched videos show promise for increasing engagement but require intentional instructional design tailored to learning objectives.

## Graphical abstract

Here, the potential of interactive, branched‐based videos in online biology courses is evaluated. Although not directly enhancing learning gains, these videos are shown to be engaging and are ideal for revision. When properly scaffolded, they excel in engaging students in problem‐solving tasks. A balance of interactivity, organisation and design of such innovative formats can enrich online learning experiences.

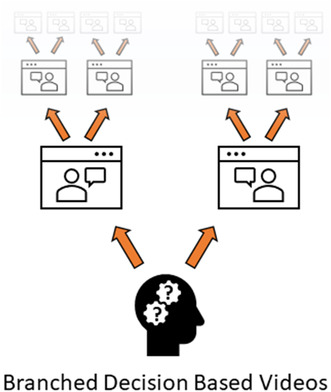

**Abbreviations**
AmS: ammonium sulphate
COVID‐19: coronavirus disease 2019
CTML: cognitive theory of multimedia learning
ER: endoplasmic reticulum
IEX: ion exchange chromatography
SEC: size exclusion chromatography

The use of asynchronous video content, in higher education teaching, has rapidly expanded since early 2020. Content has been provided as prerecorded videos for use in flipped learning activities, as lecture capture or as follow‐up consolidation learning materials [1, 2], such as short, chunked extracts covering key concepts. The use of recorded material, particularly lecture capture, is popular with students [2], and consideration of the implementation of these resources to ensure effective and engaging learning has been conducted [3, 4]. Benefits around reduced anxiety [5], language [6] and support for students with learning disabilities [7] have all been reported.

The use of videos as a learning tool complements the theoretical basis of dual coding theory, which sets out that our brains process visual and auditory information through separate systems. By engaging both these systems simultaneously, learners can better grasp and retain complex concepts. Additionally, video content can complement students' learning by allowing them to work at their own pace, review material at several points in their learning journey and reduce extraneous load, which can otherwise overload the working memory capacity and reduce the ability of the students to remember new material [8]. In 1969, Paivio [9] presented his dual coding theory, proposing that our brains have distinct but linked regions for processing visual and auditory information. Dual coding suggests that the maximal cognitive learning benefits occur when complementary information is presented simultaneously to both systems, such as occurs in well‐designed videos [9, 10]. As well as potentially helping students learn the material more efficiently, video lectures offer additional benefits to students, which centre around control over their learning. The ability to pause and rewind videos allows students to manage their cognitive load, specifically the intrinsic load of the task, which is defined as the load placed on the working memory inherent to the task itself [11]. There are also benefits to the academic, being able to edit prerecorded videos allowing for a reduction in extraneous load [12], which is where the working memory becomes ‘distracted’ by content that is not inherent to the learning outcome [11]. Through editing, academic staff can limit these extraneous details that could otherwise overload the working memory capacity and reduce the ability of the students to remember new material [13, 14].

This is not to say that videos are without drawbacks; one of the biggest challenges faced by academics is retaining student interest. Distant learning environments have been linked to waning subject interest and motivation among students due to feelings of isolation online [15, 16]. Traditional video lectures offer a very linear learning experience, and many students will not be actively engaged while watching a video recording, with the level of student learning having been suggested as proportional to the degree of interactivity in a learning resource [17]. Interactivity is more easily facilitated in small group environments where students can work together, such as an online synchronous breakout room or an in‐person seminar environment. As a result, asynchronous videos often offer fewer learning opportunities than synchronous classes.

The cognitive theory of multimedia learning (CTML) is a well‐established principle in the field of education that emphasises the importance of video instruction [4]. The theory is based on three fundamental assumptions, as discussed previously. The first assumption is the dual‐channel assumption, which asserts that people have separate information processing channels for visual and auditory information. The second assumption, called the limited processing capacity assumption, suggests that people have a limited amount of working memory available for processing information. Finally, the active processing assumption posits that learning is most effective when learners are actively engaged in the learning process.

According to CTML, video instruction should aim to reduce extraneous cognitive load, which refers to any information that is not relevant to the learning task, while increasing germane cognitive load, which refers to the cognitive processing that directly contributes to learning. In practical terms, this means that video instruction should be designed to present information in a clear and concise manner, with minimal distractions or unnecessary elements. By doing so, learners are able to focus their attention on the most important aspects of the content, which can enhance their understanding and retention of the material. This theory hence advocates for an instructional strategy that is engaging and cognitively stimulating, akin to the concept of active learning.

Active learning is an instructional approach that empowers the learner and pushes them to take control of their learning process, instead of passively receiving instruction [18]. Rather than merely listening to or watching video content, active participation entails full engagement with the recording, typically through problem‐solving activities or discussions [19]. This student‐centred strategy requires students to own their learning experiences [20]. Such learner empowerment has been underscored in numerous studies, which have shown its various benefits such as reduced failure rates [21], improved grades [22] and enhanced knowledge retention [23]. Thus, the active processing assumption of CTML aligns well with the principles and demonstrated benefits of active learning.

Introducing learner empowerment through videos has traditionally been done through interactivity, such as embedding quizzes using technology such as Panopto (Seattle, WA, USA) with an associated increase in subsequent attainment [24]. An additional innovative approach to enhancing interactivity in videos is integrating adaptive assessment or delivery, which dynamically adjusts to the learner's performance and knowledge gaps [25]. This enables a personalised learning experience, catering to individual needs and promoting greater engagement.

We postulate that branched, decision‐making videos will aid in learner understanding of key content and provide an engaging mechanism to interact with the video content. Here, we are developing, delivering and evaluating video content that allows students to determine their own path through the taught video‐based material via a branched decision tree, with each choice leading to a different outcome, which may include unexpected consequences, such as an experiment failing. By incorporating branched, decision‐making videos, the delivery is adapted and guided by the student's current understanding of the material. This fosters a learning environment that is interactive and responsive to the learner's progress.

We propose that by introducing interactivity into online, asynchronous video content using branched, decision‐making videos that students will become more engaged in the learning experience.

## Materials and methods

### Decision‐making videos

**Fig. 1:**
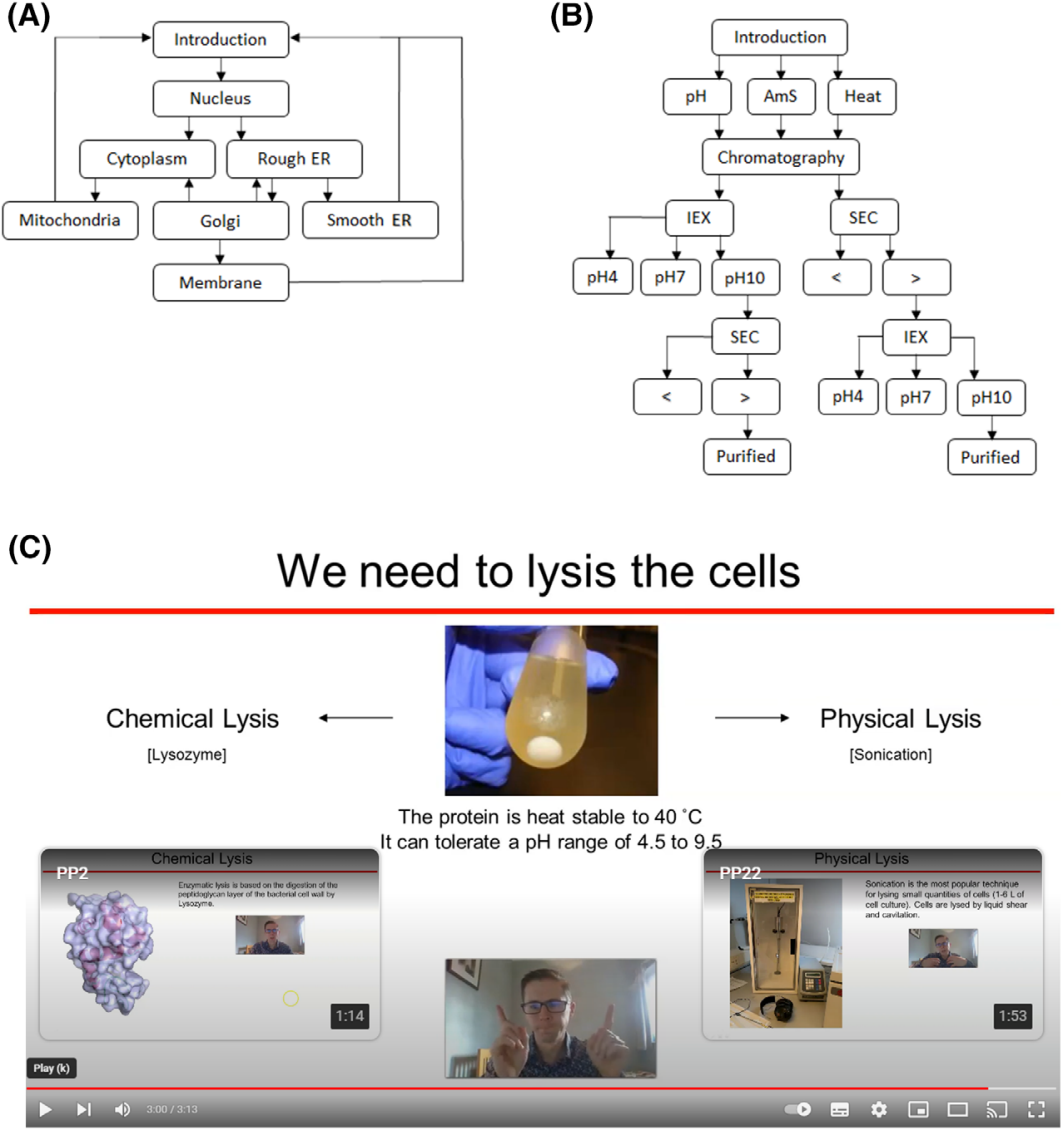
Schematic diagram of branched decision‐making videos. (A) Eukaryotic cell biology video plan. (B) Protein purification video plan. Boxes indicate individual videos with arrows indicating the students' choice of which video to view next. AmS, ammonium sulphate; ER, endoplasmic reticulum; IEX, ion exchange chromatography; SEC, size exclusion chromatography, < >, choice of matrix. (C) Screenshot example from the YouTube video showing the choices given to the student at the end of a short video (https://youtu.be/moHpXAfjhqc).

Decision‐making videos were designed to give students a choice in what to watch next while working through a given scenario. Videos were hosted on YouTube, and each component video was short, between 50 s and 2 min. Figure 1A shows the eukaryotic cell biology decision tree, whereby students could select which organelle to move to next. Figure 1B shows the protein purification problem‐solving storyboard where, at each stage, the students were presented with the option of what experiment to perform next in the purification procedure and/or the condition under which that experiment would be performed. At each stage, the students make their choice by clicking a video‐embedded URL at the end of each video. To achieve this, the End Screen function in the YouTube video editor was used and linked to the next video in the storyboard (e.g. Fig. 1C).

Within the study, a comparative, passive ‘linear video’ was created by editing the short videos into a continuous stream, removing the decision‐making aspect, yet covering the same learning materials.

### Data collection

Participants were invited to join the study through their online learning materials and were asked to consent to the research. Videos were embedded in the students' learning materials in the form of a closed Google Forms digital work booklet hosted on the University's Google Apps provision. These digital workbooks were embedded in the VLE and hosted the videos and multiple‐choice questions. Firstly, all students attempted a set of knowledge‐based multiple‐choice questions and then watched either the decision‐based or the linear video. After interacting with the video, the students completed the same set of knowledge‐based multiple‐choice questions as found at the start of the workbook. Students were then presented with the alternative video for comparison. In this way, all students gained access to the same material and what differed was the initial mode of delivery ensuring equity in the student learning experience. Student feedback on the interactive decision‐making video was then obtained through Likert scale, multiple‐choice and open‐text questions. Student email addresses were collected upon completion of the work booklet. These email addresses were replaced with a unique number and the date and time of submission were used to determine the student's first attempt, which was used for data analysis. Data collection occurred 2 months after the initial release of the learning materials, and the data were anonymised and stored securely. All raw data are available on request in line with the fair usage policy of FEBS OpenBio.

### Participants

An active participant is defined as one who attempted their assessments within the module the study was situated. Participants needed to opt into the study within 2 months of the learning materials being released. In all cases, students were randomly allocated the interactive decision‐making learning materials, or the passive, linear learning materials.

#### Cohort 1: Cellular biology self‐guided learning

Students who engaged with the eukaryotic cell biology videos were first‐year undergraduate Biosciences students. The learning material and activity sat within a 20‐credit yearlong module. Cohort 1 data were collected in 2020–2021. From a cohort of 238 active participants, 113 students (49%) engaged in the videos and opted into the study. Fifty‐seven were allocated the interactive material, and 54 the linear material. These videos were given as self‐guided learning material.

#### Cohort 2: Cellular biology consolidation material

The cell biology videos created for Cohort 1 were used for the next cohort of first‐year undergraduate Biosciences students as consolidation material after a 30‐min online lecture on the topic. The learning material and activity sat within a 20‐credit yearlong module. Cohort 2 data were collected in 2021–2022. From a cohort of 185 active participants, 49 students (26%) engaged in the videos and opted into the study. Twenty‐three were allocated the interactive material, and 26 the linear material.

#### Cohort 3: Protein purification consolidation material

Students who engaged with the protein purification videos were second‐year undergraduate and MSc Pharmaceutical Biotechnology students. The learning material and activity sat within a 20‐credit yearlong module for second‐year students and a 15‐credit semester‐one module for Masters students. Cohort 3 data were collected in 2020–2022. From a cohort of 120 active participants, 74 students (61%) engaged in the videos and opted into the study. Forty‐six were allocated the interactive material, and 30 the linear material. These videos were used as consolidation material and given to students after the initial core concepts were explained.

### Data analysis

Questions designed to test understanding were asked before and after the students' interaction with the learning material. These were then scored, with each correct answer being given 1 mark and each participant given pre‐ and post‐learning materials scores. These were then compared for each learning material type and the overall increase in learning was determined and compared between the video types.

Within the evaluation, the students were asked to ‘describe the [decision‐based video learning] experience in three words’. The student responses were thematically analysed and the frequency with which each theme occurred in the dataset was determined. Students' perceptions of the decision‐making video were collected using Linkert scale questions; answers were converted to numbers from 1 to 5 for analysis.

Statistical analysis was undertaken in Stats Direct with Wilcoxon signed rank for nonparametric paired data, Mann–Whitney for nonparametric unpaired data, Kruskal–Wallis for nonparametric data with all pairwise comparisons via Conover–Iman and chi‐squared for nominal data, where appropriate, with a *P*‐value of less than 0.05 deemed to be significant.

### Ethics

Ethics for this study was acquired through the School of Health, Wellbeing and Life Sciences ethics committee following the Sheffield Hallam University Research Ethics Policy (reference: ER26758337). Ethics approval was given as no identifiable, confidential or controversial information would be collected. No gender, age, other educational experience or other demographic factors were requested or considered within the analysis, primarily to ensure the questionnaire was concise and the length was not a barrier to completion. Participation in the study was optional, and the students registered their written consent by responding to the question below, which was presented after the university's standard ethical research statement.

I opt into the study and consent for my answers from my pre and post‐video quizzes to be analysed along with my answers to the evaluation. YES/NO.

## Results

The purpose of this study was to investigate whether interactive, decision‐making videos could improve students' understanding and engagement with prerecorded video‐based learning materials. The learning material was presented in an online work booklet, consistent with the rest of the module. Students in the study were randomly assigned to one of two groups; one group received the interactive, decision‐based learning material while the other group received the linear, passive learning materials. To measure students' understanding of the learning material presented in the videos, a series of questions related to knowledge and understanding were asked both before and after watching the videos. A follow‐up evaluation of the interactive, decision‐based learning content was conducted.

### Branched and linear videos increase knowledge

**Fig. 2:**
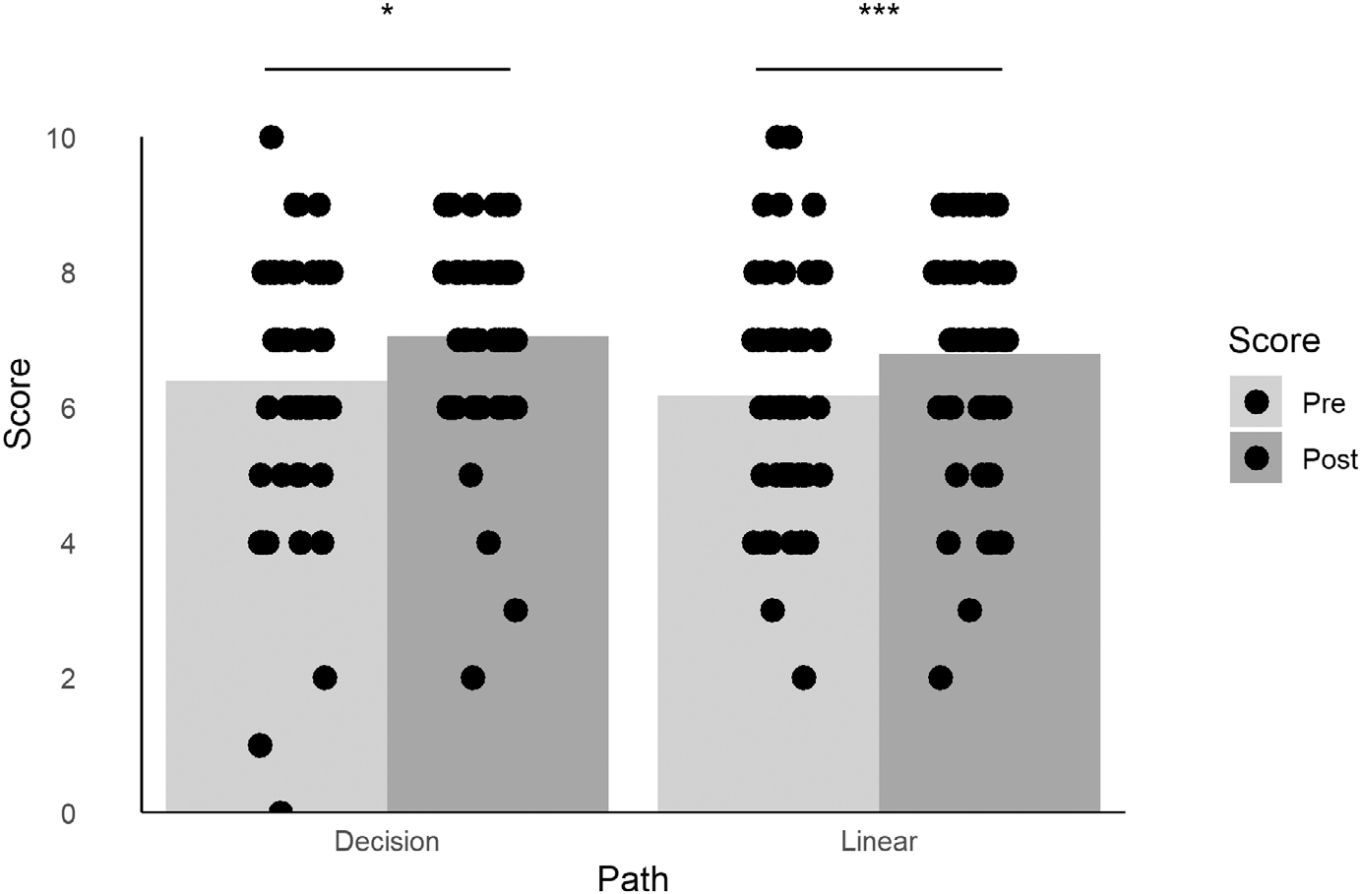
Cohort 1 knowledge before and after the first engagement with decision‐based or linear videos. Each point represents an individual student score, where more than one student gained the same score; this has been shown using a Bee Swarm visualisation. Data shown are the mean. * indicates *P* < 0.05; ***P < 0.001 in a Wilcoxon signed rank test (decision *n* = 51, linear *n* = 54).

Initially, online learning materials were designed for first‐year Biosciences undergraduates as self‐guided learning material on eukaryotic cell biology (Cohort 1). Students' understanding was determined before and after interaction with the learning material (Fig. 2).

Students increased their knowledge with either linear or decision‐based online content. There was no statistically significant difference between the pretest scores or the overall learning gains between the types of delivery (*P* = 0.8559 in a Mann–Whitney *U*‐test).

### Interactive learning videos in self‐guided learning–students' experiences (Cohort 1)

After completion of the post‐learning material questionnaire, the participants were invited to engage with the alternative learning material. Participants were asked what three words described their experience interacting with self‐guided decision‐based videos. These were blinded and a thematic analysis was undertaken (Table 1: Cohort 1).

**Table 1:** Thematic analysis of participants' experience of interacting with decision‐based videos. Data shown are the number of times each theme was identified in participants' responses. Percentages were calculated by the number of responses in a theme within a cohort. Note: as participants' responses may include more than one theme, the percentages are therefore over 100% in total. Cohort 1 *n* = 93, Cohort 2 *n* = 20, Cohort 3 *n* = 37.

Theme	Cohort 1: Cellular biology self‐guided learning	Cohort 2: Cellular biology consolidation material	Cohort 3: Protein purification consolidation material	Example response
Enjoyable	44 (47%)	14 (38%)	10 (50%)	‘Fun’, ‘enjoyable’, ‘great’
Positive platform	49 (53%)	14 (39%)	7 (35%)	‘Engaging’, ‘simple’, ‘easy to follow’
Positive learning experience	42 (45%)	18 (48%)	11 (55%)	‘Informative’, ‘effective’, ‘interesting’
Not enjoyable	8 (9%)	6 (16%)	0	‘Boring’, ‘overwhelming’, ‘anxiety’
Negative platform	11 (11%)	4 (8%)	0	‘Time‐consuming’, ‘laborious’, ‘disorganised’
Negative learning experience	17 (18%)	5 (11%)	0	‘Confusing’, ‘hard’, ‘unhelpful’

Cohort 1 student feedback on the decision‐based learning content was predominantly positive with most comments aligning to the themes of ‘enjoyable’, ‘positive platform’ and ‘positive learning experience’. Some students reported negative elements of the decision‐based learning material, with a minority reporting self‐guided interactive videos to be confusing or unhelpful. These were grouped into themes of ‘not enjoyable’, ‘negative platform’ and ‘negative learning experience’. There was no statistical difference in the thematic analysis between those initially allocated the decision‐based video and those who viewed it after the linear video (*P* = 0.943 in a chi‐squared test). To evaluate the effectiveness of decision‐based learning material evaluation, students were asked ‘I would like more interactive videos in the future’ (Table 2: Cohort 1).

**Table 2:** Total frequency of students' willingness for more interactive videos in the future. Cohort 1 *n* = 120, Cohort 2 *n* = 44, Cohort 3 *n* = 30.

‘I would like more interactive videos in the future’	Cohort 1: Cellular biology self‐guided learning	Cohort 2: Cellular biology consolidation material	Cohort 3: Protein purification consolidation material
Yes	46 (38%)	16 (36%)	22 (74%)
Maybe	53 (44%)	22 (50%)	7 (23%)
No	21 (18%)	6 (14%)	1 (3%)

Within Cohort 1, there was no difference between those who initially undertook the decision‐based or linear video in their willingness for more interactive videos (*P* = 0.423 in a chi‐squared test). Students in Cohort 1 were overall positive, with 82% saying ‘yes’ or ‘maybe’, about engaging with interactive videos in the future.

**Fig. 3:**
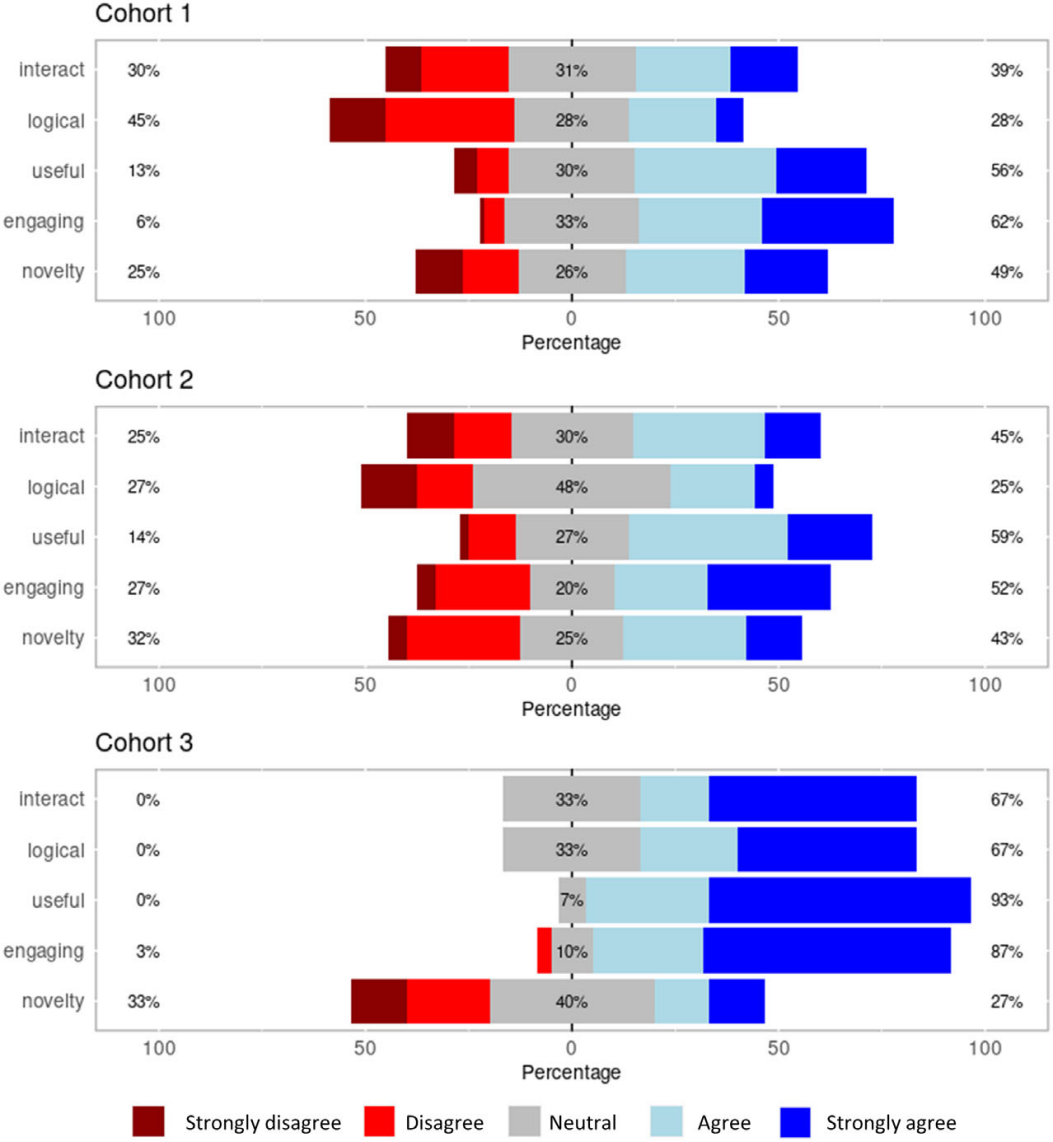
Students' perceptions of decision‐making learning material. Interact: ‘I could interact with the decision‐making videos easily’, logical: ‘The decision‐making video material was presented in a logical manner’, useful: ‘I found the decision‐based videos more useful for learning than linear videos’, engaging: ‘I found the decision‐based videos more engaging than linear videos’, novelty: ‘The decision videos were engaging but the novelty would soon wear off’. Cohort 1 *n* = 104, Cohort 2 *n* = 44, Cohort 3 *n* = 30. Percentages on the left side represent the total of strongly disagree and disagree, within the middle grey section neutral, and on the right side the total of agree and strongly agree.

Participants were then asked a series of questions about their perceptions of decision‐making online learning material. Responses were rated on a Likert scale between 1 (strongly disagree) and 5 (strongly agree; Fig. 3: Cohort 1).

Within Cohort 1, less than half (39%) of participants agreed with the statement that the decision‐making videos are easy to interact with and 28% agreed that they were presented in a logical manner; however, the majority of students (62%) agreed that they were more engaging with 56% agreeing that they were more useful than linear videos. There were no differences between those who initially undertook the decision‐based or linear video (‘I found the decision‐based videos more engaging than linear videos’ *P* = 0.283; ‘I found the decision‐based videos more useful for learning than linear videos’ *P* = 0.669; ‘The decision making video material was presented in a logical manner’ *P* = 0.141; ‘I could interact with the decision‐making videos easily’ *P* = 0.503; ‘The decision videos were engaging but the novelty would soon wear off’ *P* = 0.639 in Mann–Whitney tests).

Finally, participants were asked if they had any comments on the use of decision‐making videos in teaching, this was a free text response. Participants commented that there needed to be a clearer structure to the decision‐making video content:

“My only concern is that I may have missed some of the videos, depending on the choices I made.”

“Felt like the non‐linear nature made me more concerned I'd missed part of the lecture rather than focusing on what was being presented.”

“It made me anxious that I was going to miss out on content. I worried about accidentally missing out on a video from never having clicked it.”

This concern regarding missing content was echoed throughout other comments where students suggested the use of decision‐based videos for revision or topics that are difficult to engage with.

“I think the non‐linear style could be good for revision.”

“Some topics it's more engaging to use e.g. heavy content stuff that needs a lot of basic understanding.”

“Decision‐making vids are better for taking in the info in a revision sense, but linear vids are better when the content is better off being taught in a certain order.”

### Interactive learning videos as consolidation material (Cohorts 2 and 3)

Based on the analysis of Cohort 1 and the suggestion by participants that interactive learning videos would be a suitable consolidation activity, the eukaryotic cell biology decision‐based learning material was made available as consolidation material to Cohort 2 the following year. In the cell biology session for Cohort 2, the lecture material was initially delivered, as with the rest of the module content, online and the interactive learning videos were given to the students afterwards as consolidation material. The same recruitment and evaluation methodology was used as for Cohort 1.

Students had a similar experience of interacting with the decision‐based videos based on thematic analysis (Table 1: Cohorts 1 and 2) as Cohort 1. Interestingly, no student in Cohort 2 mentioned ‘overwhelming’ or ‘anxiety’ and negative comments were about it being time‐consuming or confusing. There was no statistical difference between the frequency of themes in Cohort 1 and Cohort 2 (*P* = 0.551 in a chi‐squared test). In addition, students in Cohort 2 had a similar willingness to engage with more decision‐based videos as in Cohort 1 (Table 2: Cohorts 1 and 2). There was no difference in student responses between those who completed the decision‐making material first and those given access afterwards (*P* = 0.464 in a chi‐squared test).

Subsequently, participants in Cohort 2 were asked about their perceptions of decision‐making online learning material (Fig. 3: Cohort 2). There was no significant difference in students' responses between Cohorts 1 and 2 and any of the questions (‘I found the decision‐based videos more engaging than linear videos’ *P* = 0.992; ‘I found the decision‐based videos more useful for learning than linear videos’ *P* = 0.613; ‘The decision making video material was presented in a logical manner’ *P* = 0.950; ‘I could interact with the decision‐making videos easily’ *P* = 0.083; ‘The decision videos were engaging but the novelty would soon wear off’ *P* = 0.651 in Mann–Whitney tests) and responses remained broadly positive.

The initial cell biology learning materials were presented to students with the learning objective of establishing core knowledge of cell biology. Subsequently, we wanted to determine whether interactive decision‐based videos could be used for problem‐solving activities. To achieve this, self‐directed learning materials were created to consolidate learning around protein purification, where students made choices around an experimental workflow. Cohort 3 was second‐year Biosciences undergraduates and MSc Pharmaceutical Biotechnology students.

Protein purification decision‐making videos were presented to Cohort 3 through digital workbooks in the same manner as Cohort 2. On completion of the workbook, thematic analysis of students' experience of interacting with decision‐based videos was conducted through the same open‐text questions as Cohorts 1 and 2. The analysis identified no negative comments associated with the learning material (Table 1: Cohort 3). When participants were asked, ‘I would like more interactive videos in the future’ 74% stated ‘yes’ (Table 2: Cohort 3). This willingness to engage in decision‐making videos was statistically higher than those in Cohorts 1 and 2 (Table 2; *P* < 0.05 in a chi‐square test).

To determine why students in Cohort 3 were more willing to engage in decision‐based videos, a comparison of participants' perceptions of decision‐making online learning material between the three cohorts was undertaken (Fig. 3). Ninety‐three per cent of these students responded that decision‐based videos were more useful for learning than linear video; however, no significant learning gains were identified within the cohort over their linear delivery counterpart (*P* = 0.440, Wilcoxon signed rank test). Cohort 3 showed a significant increase in positive responses compared with Cohorts 1 and 2 in the questions ‘I could interact with the decision‐making videos easily’; ‘The decision‐making video material was presented in a logical manner’; ‘I found the decision‐based videos more engaging than linear videos’ and ‘I found the decision‐based videos more useful for learning than linear videos’ (*P* < 0.01, Kruskal–Wallis: all pairwise comparisons via Conover–Iman). There was no difference between cohorts to the question ‘The decision videos were engaging but the novelty would soon wear off’. The data analysis demonstrates that branched decision‐based videos are an effective way of delivering problem‐solving activities in a self‐directed digital environment.

## Discussion

Educational videos are known to be effective teaching resources in online courses; however, students often have difficulty sustaining their attention while watching these videos and subsequently comprehending the content [26]. Targeted YouTube videos have been shown to enhance student engagement, depth of understanding and overall satisfaction in higher education courses [27], with fun and interactive activities increasing critical thinking skills [28]. In the present study, we aimed to evaluate the efficacy and student perception of interactive, decision‐making videos compared with traditional linear videos in an online learning context. The results of this investigation provide significant insights into the potential advantages around engagement and limitations of these two modes of video‐based learning. Such approaches have been described as adventure learning [29]. This learning model emphasises active, authentic and participatory learning, engaging students in exploring real‐world issues through narrated, online and collaborative experiences. Students participating in such activities are not merely passive recipients of information, instead, they are active collaborators, who participate in problem‐solving tasks [30].

Although the decision‐based videos did not increase learning gains, they may hold the students' attention and increase engagement in blended activities over longer periods and it has been suggested that time spent watching is a better metric of engagement [31]. It is important to note, therefore, that the design of the intervention has a limitation: the branched nature of the videos makes it difficult to determine the exact amount of time spent watching. This is due to the looped nature of the videos, and as a result, we have no way of directly determining the individual learning paths. However, since the branches only occur at the end of the video, students must have watched the entire video to continue their learning journey. In a separate study of remote learner engagement, digital videos were shown to make a substantial contribution to subject engagement if the videos are relevant, concise and interesting, allowing for interaction [32]. Giannakos *et al*. [33], in their investigation of video usage in students, concluded that video usage styles can significantly impact student engagement, with active and creative video usage styles being associated with higher levels of engagement. Similarly, Choi [34] argues that students who watched the lecture video with entertainment techniques, such as storytelling, were more engaged and had better learning outcomes than the students who watched the lecture video without entertainment techniques.

The design of the interactive videos in this study was such that it allowed students to direct their learning journey by deciding what video content to engage with next. This element of choice and autonomy in the learning process was positively received by a majority of students, which is consistent with previous literature [35, 36] indicating that autonomy and active involvement in video content can enhance motivation and engagement. However, it is noteworthy that when completely self‐guided videos were initially used (Cohort 1), only 56% of students found the approach more beneficial for learning when compared to the conventional linear format. The discrepancy between the enjoyment derived from the interactivity of the videos and the perceived educational utility points towards constraints in the application for core learning. Comments around the fear of missing out on critical content were the basis of this observation and scaffolding around the interaction is needed, with core content presented clearly. Interestingly, they were received more favourably when the interactive videos were adapted to offer a problem‐solving approach as consolidation material following explanation in a linear manner (Cohort 3). Ninety three per cent of students found these revised interactive videos more helpful in learning than the linear counterpart (Fig. 3, Cohort 3). Using videos as support material has been shown in other studies to improve initial learning and reduce dropout rates [37, 38, 39]. Videos have also been shown to be beneficial as a preparative activity for laboratory work [30]. Branching videos could also be used to supplement and support expensive laboratory practicals, or those requiring highly specialised equipment [30]. Their value, in this regard, is a subject of further investigation. Together, this indicates that the structure of the video content, rather than the interactive element itself, could be a key factor influencing the perceived educational value, underscoring the importance of thoughtful instructional design in creating effective learning materials [34, 40].

On reflection of decision‐based video material in practice, with regard to the time taken by the authors to make these decision‐based video resources compared with more traditional materials, it is important to acknowledge that these resources are more time‐intensive to create. However, as the data presented here shows, the student cohorts broadly appreciate the decision‐based videos, which can be used across multiple student groups and cohorts. In addition, within a classroom setting, they have high value when combined with previous delivery as a means of consolidation. Thus, the videos may be time‐intensive initially, but in the long term, they are a valuable resource. In conclusion, this study provides initial evidence supporting the utility of interactive decision‐making videos in online learning. These videos can potentially increase student engagement and enhance the perceived learning benefits, as indicated by the response to the Likert data (Fig. 3). However, the structure and organisation of these videos appear to be critical elements, and there is a need to ensure that the nonlinear nature of the interactive format does not inadvertently obstruct the learning process. Future research should further explore the potential of this innovative instructional approach, focusing on finding the optimal balance between interactivity, structure and educational impact. This could lead to more effective and engaging online learning experiences that could better cater to student's diverse needs and preferences.

## Conflict of interest

The authors declare no conflict of interest.

### Peer review

The peer review history for this article is available at https://www.webofscience.com/api/gateway/wos/peer‐review/10.1002/2211‐5463.13767.

## Author contributions

DPS, NJF and MML conceived and designed the project. DPS, NJF and MML acquired the data. DPS, NJF and MML analysed and interpreted the data. DPS, NJF and MML wrote the paper.

## Data Availability

The data that support the findings of this study are available on request from the corresponding author. The data are not publicly available due to privacy or ethical restrictions.
